# Nucleotide, Cytogenetic and Expression Impact of the Human Chromosome 8p23.1 Inversion Polymorphism

**DOI:** 10.1371/journal.pone.0008269

**Published:** 2009-12-14

**Authors:** Nina Bosch, Marta Morell, Immaculada Ponsa, Josep Maria Mercader, Lluís Armengol, Xavier Estivill

**Affiliations:** 1 Genetic Causes of Disease Group, Genes and Disease Programme Center for Genomic Regulation (CRG-UPF), Barcelona, Catalonia, Spain; 2 CIBER en Epidemiología y Salud Pública (CIBERESP), Barcelona, Catalonia, Spain; 3 Departament de Biologia Cel·lular, Fisiologia i Immunologia, Facultat de Medicina, Universitat Autònoma de Barcelona, Catalonia, Spain; 4 Institut de Biotecnologia i Biomedicina, Universitat Autònoma de Barcelona, Barcelona, Catalonia, Spain; 5 Quantitative Genomic Medicine Laboratories (qGenomics), Barcelona, Catalonia, Spain; 6 Department of Health and Experimental Life Sciences, Pompeu Fabra University (UPF), Barcelona, Catalonia, Spain; Duke University, United States of America

## Abstract

**Background:**

The human chromosome 8p23.1 region contains a 3.8–4.5 Mb segment which can be found in different orientations (defined as genomic inversion) among individuals. The identification of single nucleotide polymorphisms (SNPs) tightly linked to the genomic orientation of a given region should be useful to indirectly evaluate the genotypes of large genomic orientations in the individuals.

**Results:**

We have identified 16 SNPs, which are in linkage disequilibrium (LD) with the 8p23.1 inversion as detected by fluorescent in situ hybridization (FISH). The variability of the 8p23.1 orientation in 150 HapMap samples was predicted using this set of SNPs and was verified by FISH in a subset of samples. Four genes (*NEIL2*, *MSRA*, *CTSB* and *BLK*) were found differentially expressed (p<0.0005) according to the orientation of the 8p23.1 region. Finally, we have found variable levels of mosaicism for the orientation of the 8p23.1 as determined by FISH.

**Conclusion:**

By means of dense SNP genotyping of the region, haplotype-based computational analyses and FISH experiments we could infer and verify the orientation status of alleles in the 8p23.1 region by detecting two short haplotype stretches at both ends of the inverted region, which are likely the relic of the chromosome in which the original inversion occurred. Moreover, an impact of 8p23.1 inversion on gene expression levels cannot be ruled out, since four genes from this region have statistically significant different expression levels depending on the inversion status. FISH results in lymphoblastoid cell lines suggest the presence of mosaicism regarding the 8p23.1 inversion.

## Introduction

Among the different classes of structural variations, the understanding of the prevalence and spectrum of the inversions in the human genome is still scarce. One of the reasons is that most genome-wide technologies used to discover structural variations are designed to detect gains and losses of genomic material. Only recently, several studies based on fosmid cloning and paired-end sequencing have succeeded in mapping inversion breakpoints in a genome-wide fashion [1]–[4]. As revealed by these and other studies, which include comparing genomes assembled from different individuals and targeted analyses, the total number of inversion regions in the human genome is now close to 500 (http://projects.tcag.ca/variation/), but this figure will likely increase with deep sequencing data coming from the 1000 genomes project (www.1000genomes.org).

An indirect approach to delineate human inversions, which was first described on *Drosophila* studies [5], is the characterization of extended blocks of linkage disequilibrium (LD) that are created due to the lack of recombination in heterozygous individuals [6]. Following this criterion, haplotype subgroups can be defined in polymorphic inversions because different alleles are maintained in the different orientations. Such is the case for the polymorphic inversion on chromosome 17q21.31, for which one of the haplotypes associated to one of the conformations has been found to be under positive selection in Europeans [7], [8].

Human chromosome 8p23.1 encompasses a 3.8–4.5 Mb polymorphic segment flanked by two large blocks of segmental duplications (SDs). The whole region extends up to 6.5 Mb, which includes the SDs and contains at least 50 genes. This inversion was first described to have a frequency of 26% in the European [9] and 27% in the Japanese [10] populations, assuming that the assembly of the reference sequence corresponds to the non-inverted conformation. However, different studies have found an increased frequency of the inversion, around 60%, in populations of European ancestry, indicating that the human reference assembly corresponds to the minor allelic orientation of the region [4], [11], [12]. Moreover, the 8p23.1 region is an intricate DNA segment flanked by two large sets of SDs, named REPP (proximal) and REPD (distal), containing several genes that vary in copy number, such as defensins or *FAM90A* [13]–[22]. The 8p23.1 region has recently been reported to also contain a high concentration of structural variants in fosmid end-sequencing experiments [4]. Thus, the sizes of the flanking SDs are variable and not fully sequenced (both are defined as gaps in the assembled genome sequences), and it seems plausible that this variability at the SDs could play a key role in the distinct rearrangements affecting the 8p23.1 region.

Although initially considered a neutral polymorphism, the 8p23.1 inversion has been found in mothers of children with an associated phenotype suffering from different rearrangements involving the 8p23.1 region. This phenomenon has also been described in the parents of children carrying other genomic disorders [23] such as Hunter syndrome [24], Williams-Beuren syndrome [25], [26], and Prader-Willi or Angelman syndromes [27].

With the aim to identify single nucleotide polymorphisms (SNPs) that could be used as surrogate markers for the 8p23.1 inversion, we performed a genotyping analysis of six individuals that were analyzed by fluorescent in situ hybridization (FISH) and were found to be homozygous for the 8p23.1 inversion (with respect to the reference genome sequence). We have identified two small tracts of SNP in homozygosity, which perfectly correlate with the inversion status of this genomic region. Thus, we predicted the genotype of the inversion for 150 HapMap individuals using the genotype of these SNPs, and we confirmed our predictions by FISH analysis in a subset of these samples. Interestingly, we have detected a variable degree of mosaicism with respect to the inversion within all the samples analyzed by FISH. This phenomenon suggests that the region is mitotically unstable. Finally, we have explored the effect of the inversion rearrangement on 8p23.1 gene expression levels and have found that four genes from this region have statistically significant different expression levels depending on the inversion status.

## Methods

### Genotype Data and the Delineation of Homozygosity Tracts

A deep SNP genotyping analysis of the 8p23.1 region was performed using the HumanCNV370-Duo chip from Illumina in a sample of six Spanish individuals of the general population that were found to be homozygous for the 8p23.1 inversion FISH, and the HapMap sample NA10861 was used as a control for genotype concordance. Among all markers covered by the array, we focused on 770 SNPs spanning the 8p23.1 region, where two tracts of homozygosity containing 16 SNPs could be defined between 8.5–8.7 Mb and 10.8–11 Mb in the six homozygous inverted individuals. Genotyping was carried out following manufacturer's protocol (Illumina Inc.) at the Barcelona CeGen genotyping center. Illumina's HumanCNV370-Duo chip includes common variation described for the CEU, CHB/JPT, and YRI populations based on HapMap Phase I and II data, and also contains probes that are enriched in CNV and SDs.

Genotype data of 210 unrelated HapMap individuals, including 60 parents of 30 trios from Utah residents with ancestry from northern and western Europe (CEU), 60 parents of 30 trio samples from Yoruba in Ibadan, Nigeria (YRI), 45 unrelated Han Chinese from Beijing, China (CHB), and 45 Japanese from Tokyo, Japanese (JPT) individuals, were also used in the analysis. Due to the significant genetic similarity between Chinese and Japanese groups, we pooled CHB and JPT data and denoted these individuals as Asian (ASN) as in other studies [28].

HapMap phase II data was downloaded from the website (HapMap Data Rel23a/phaseII Mar08; www.hapmap.org). Phased haplotypes were used to predict the genotype of the 150 individuals with respect to the 8p23.1 inversion based on the conserved homozygosity tracts previously described. The 150 individuals analyzed included the 60 parents from CEU samples and the 90 ASN individuals.

### FISH Analysis

FISH was performed on metaphase chromosomes prepared from lymphoblastoid cell lines obtained from 24 unrelated Spanish individuals using BAC-derived DNA probes. Two BAC clones that fall within the 8p23.1 inversion and that are free of SDs (RP11-399J23 and RP11-589N15) were used for the experiments. DNA from BAC clones was isolated by standard procedures and labeled with Spectrum green for RP11-399J23, and with Spectrum orange for RP11-589N15. Labeled probes were precipitated and resuspended following standard protocols. Metaphase chromosomes and probes were denatured and hybridized overnight at 37°C. Chromosomes were counterstained with 4′, 6-diamino-2-phenylindole (DAPI). Images were analyzed using the Isis software from Metasystems. At least 20 metaphases with clearly interpretable signals on both chromosomes were counted per individual.

Elongated chromosomes were obtained adding 10 µl of bromodeoxyuridine (10 mg/ml) for three hours incubation time, followed by a 75 minutes incubation time with 10 µl of ethidium bromide (10 mg/ml). This treatment was used for individuals LCL 345, LCL 247, LCL 384, LCL 339, LCL 221, LCL 137, LCL 342, LCL 340, LCL 198, LCL 179, LCL 182, LCL 240 and LCL 195.

HapMap cell lines were obtained from Coriell Repositories (Camdem, NJ). FISH analysis following the same procedure was performed in 9 HapMap samples to confirm the predictions for the 8p23.1 inversion based on the described surrogate SNP markers. Individuals NA11992, NA12057 and NA11839 were used for confirmation of the non-inverted orientation of 8p23.1. NA11993, NA06993 and NA11994 were the heterozygous individuals. Finally, the homozygous status of the inversion was confirmed in individuals NA11831, NA12815 and NA12155.

### Association Study of 8p23.1 Inversion and Gene Expression Levels

Normalized gene expression values from CEU and CHB/JPT (ASN) were downloaded from the Sanger Genevar webpage (http://www.sanger.ac.uk/humgen/genevar/). Data retrieved by 31 Illumina™ different probes targeting 26 genes located around the 8p23.1 region (Table S2) were used to explore the correlation between gene expression levels and the three different genotypes of the inversion region (non-inverted homozygous, heterozygous, and homozygous inverted). Individuals included in this analysis were the 150 samples corresponding to the CEU and ASN populations where the status of the inversion could be predicted using surrogate markers. The association between gene expression values and the genotype for the inversion was tested for different genetic models. P-values were derived from likelihood ratio tests, and a significance level of 5% (two sided) was used for the analyses. All these analyses were performed using the SNPassoc R package [29].

## Results

### Frequency of 8p23.1 Inversion in the Spanish Population

To determine the frequency of 8p23.1 inversion in the Spanish population, we genotyped a total of 24 Spanish control individuals by FISH. We used BAC clones RP11-399J23 and RP11-589N15, localized at each end of the 3.8 Mb inversion on chromosome 8 and outside the SDs flanking 8p23.1 [10]. Based on the human reference assembly Human NCBI Build 36 we consider the telomere-to-centromere orientation RP11-399J23 (Green) and RP11-589N15 (Red) as the non-inverted conformation. The hybridizations were performed using several slides per individual. For 13 of the 24 individuals we obtained elongated and non-elongated metaphase chromosomes.

**Figure 1:**
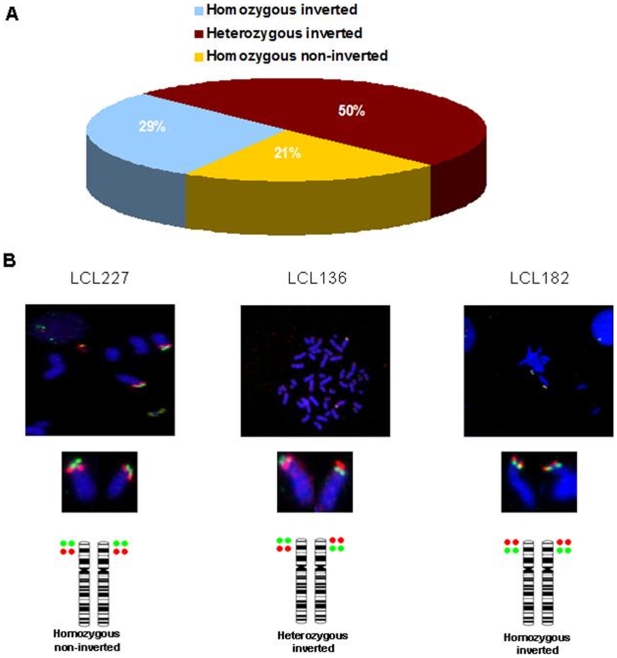
FISH analyses for the human chromosome 8p23.1 inversion in control samples. DNA probes were generated from BAC clones RP11-399J23 (Green) and RP11-589N15 (Red). A Frequencies of each of the three genotypes for the 8p23.1 inversion obtained in 24 Spanish control samples. B Metaphase FISH of three of these samples, LCL227 is “Build-36-non-inverted”; LCL136 is heterozygous for the 8p23.1 inversion, and LCL182 corresponds to homozygous “Build36-inverted”.

From the 24 individuals that were genotyped, 50% (N = 12) were heterozygous for the inversion, 29% (N = 7) homozygous for the inverted conformation, and 21% (N = 5) homozygous for the Build 36 genome assembly conformation (Figure 1). Thus, the 8p23.1 inversion was found in ∼80% of control individuals and consequently the reference assembly exemplifies the less frequent orientation of this genomic segment in the Spanish population.

The study also revealed that although a predominant genotype could be established for each studied sample, metaphases corresponding to different genotypes were observed in the 24 individuals analyzed (a subset of these samples is shown on Table 1). Therefore, from the 12 samples heterozygous for the inversion, the percentage of metaphases homozygous for either the normal or the inverted conformation ranged from 14% to 38%. Among the seven individuals homozygous for the inversion, 4% to 32% of the metaphases resulted homozygous for the non-inverted conformation or heterozygous for the inversion. Finally, among the 5 individuals that were found to be homozygous for the non-inverted conformation, up to 18% of the metaphases had a heterozygous genotype.

**Table 1:** FISH analysis of 8p23.1 inversion in Spanish control individuals.

	METAPHASES		
Samples	Homozygous non-inverted	Heterozygous	Homozygous inverted
LCL159	87%	10%	3%
LCL183	82%	18%	-
LCL198	92%	8%	-
LCL146	14%	62%	24%
LCL161	19%	75%	6%
LCL184	13%	84%	3%
LCL241	5%	86%	9%
LCL247	20%	70%	10%
LCL194	4%	8%	88%
LCL339	-	22%	78%

Percentages of each of the three possible genotypes observed within each Spanish individual in a subset of 10 representative samples from the 24 individuals analyzed. The predominant genotype is represented as a grey box.

These results suggest instability of this genomic region, probably due to intrachromosomal recombination between the two sets of large SDs at both 8p23.1 extremes of the inversion polymorphism region. The data also highlights the difficulty when interpreting the genotype for the 8p23.1 inversion using FISH. According to our results, a predominant genotype can be assigned to each sample, but somatic mosaicism seems to be common, at least in samples derived from lymphoblastoid cell lines.

### Homozygosity Blocks in 8p23.1 Inverted Alleles

**Figure 2:**
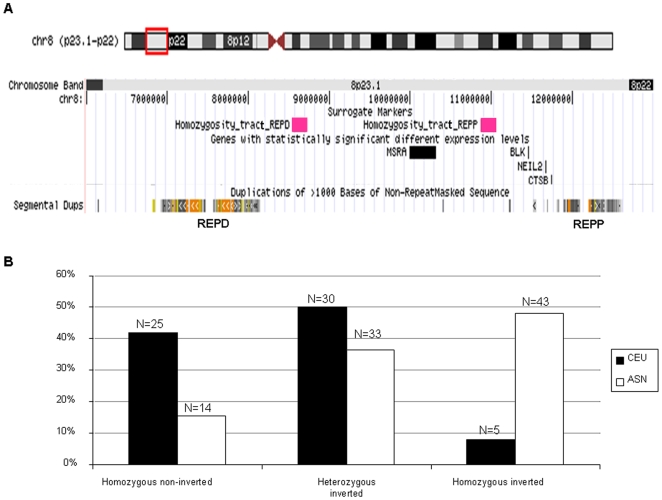
Scheme of the localization of the homozygosity tracts used as surrogate markers to predict the status of the human chromosome 8p23.1 inversion in HapMap samples (Homozygsity tract_REPD and Homozygosity tract_REPP). Genes differentially expressed on the association analysis and SDs are also depicted. B Frequencies predicted for the 8p23.1 inversion in CEU (white bars) and ASN (black bars) populations based on haplotypes for two blocks of 8 SNPs inside the region of the polymorphic inversion.

If we consider that haplotype subgroups are created by suppression of recombination in the 8p23.1 inverted fragment, we should be able to describe surrogate markers for 8p23.1 inversion. To disclose the surrogate markers, six of the Spanish control individuals, that were found to be homozygous for the 8p23.1 inversion by FISH analysis, were genotyped using the Illumina's HumanCNV370-Duo chip. Homozygous-inverted individuals were chosen as the presence of homozygosity tracts simplifies haplotype estimation and avoids phasing errors. By this procedure we were able to delineate two different homozygosity tracts composed by a total of 16 SNPs within the inverted region, in the proximity of the REPP and REPD duplicons. The first homozygosity tract expands ∼172 kb and contains 8 SNPs present in the HumanCNV370-Duo chip and corresponds to the “CGTCGAGG” haplotype in all 6 individuals (Table 2). This genetic “signature” is located at chromosome position 8.5 Mb, close to the REPD distal SDs that flank the 8p23.1 segment. A second block of 8 homozygous SNPs spans ∼181 kb, and in this case the conserved haplotype is “TCACGAGA” (Table 2) and lays at 10.8 Mb, close to REPP, the proximal set of SDs on 8p23.1 (Figure 2A).

**Table 2:** Tracts of homozygosity in 8p23.1 region extracted from the whole genome scan data performed in homozygous inverted individuals.

REPD		REPP	
SNP	Homozygous inverted haplotype	SNP	Homozygous inverted haplotype
rs17627505	C	rs1178061	T
rs10503393	G	rs1178247	C
rs2428	T	rs3885690	A
rs11774860	C	rs2409691	C
rs3827811	G	rs13266785	G
rs17154769	A	rs10282848	A
rs1876836	G	rs10503417	G
rs1039916	G	rs2409719	A

The 8 SNPs that serve as surrogate markers close to the REPD duplicons are shown on the left. The 8 SNPs that serve as surrogate markers close to the REPP duplicons are shown in the right.

Assuming that these 16 SNPs remain as a “signature” which resulted from the recurrent recombination processes that lead to the inversion of the 8p23.1 region, we postulate that these two 8-SNP haplotypes can be used as proxies for the 8p23.1 inversion. We downloaded the phased haplotypes from the 210 HapMap samples (only the parents from the CEU and YRI trios were used) to predict the status of the inversion in these samples. In the population of European ancestry we found that 50% of the individuals are heterozygous for the inversion, 8% are homozygous, and 42% do not have the inverted allele (Figure 2B). Among the population of Asiatic origin the presence of the inversion is extremely high, with 37% of the samples being heterozygous and 48% homozygous for the 8p23.1 inversion (Figure 2). Finally, these two 8-SNP haplotypes were not detected in the YRI samples. This is probably because our predictions are based on Caucasian ancestry individuals, and YRI samples might have different LD patterns and the inversion could not be tagged with these SNPs. Therefore, the profiles segregating with the inversion could not be assessed by our approach, although it has recently been reported that ∼60–76% of YRI individuals have the inversion [4], [12].

### Confirmation of the Predictions for 8p23.1 Inversion by FISH Analysis

In order to confirm the validity of the two 8-SNP haplotypes proxies for the presence of 8p23.1 inversion in Caucasian samples, we have genotyped the inversion by FISH in a subset of HapMap CEU samples. We choose three individuals predicted to be homozygous for the 8p23.1 inversion (NA11831, NA12815 and NA12155), three heterozygous (NA11993, NA06993 and NA1994) and three homozygous for the non-inverted status (NA11992, NA12057 and NA11839) (Figure S1). The predicted genotype was confirmed in the 9 samples, although again some degree of mosaicism was detected but with a predominant genotype (Table 3). These findings are in agreement with the large number of discordant fosmids per individual found in HapMap samples by Kidd et al. (2008), in the 8p23.1 region (Figure S2 and Table S1). Although the discordant fosmid clones could be due to a mapping artifact due to the presence of the SDs at both ends of the inverted region, several of the fosmid end-sequences are clearly different, unambiguously mapping to the regions reported by Kidd et al. (2008). The results reported here and the fosmid end-sequencing data suggest mitotic rearrangements leading to different population of cells regarding the orientation of the 8p23.1 polymorphic segment.

**Table 3:** FISH analysis in HapMap samples.

HapMap sample	Homozygous non-inverted	Heterozygous	Homozygous inverted
NA11992	95%	5%	-
NA12057	67%	33%	-
NA11839	97%	3%	-
NA11993	-	96%	4%
NA06933	-	91%	9%
NA11994	-	75%	25%
NA11831	-	38%	62%
NA12815	-	35%	65%
NA12155	-	46%	54%

DNA probes were made from BAC clones RP11-399J23 (Green) and RP11-589N15 (Red).

Our FISH analysis showed that in individuals NA11992, NA12057 and NA11839 the predominant genotype is the non-inverted status. Individuals NA11993, NA06933 and NA11994 have most metaphases heterozygous for the 8p23.1 inversion. In samples NA11831, NA12815 and NA12155 the most observed genotype is the homozygous inverted. We propose that the alternative conformations for the 8p23.1 region arise by intrachromosomal homologous recombination at mitosis and that these two 8-SNP haplotypes are reliable markers to infer the genomic structure of the 8p23.1 region.

### Gene Expression Analysis of 8p23.1 Genes in HapMap Populations

Another aim of this study was to investigate if the inverted conformation has an effect on the expression of the genes contained in the 8p23.1 region. For this purpose we made use of gene expression levels in lymphoblastoid cells of HapMap individuals [30] and we performed an association study with respect to the inversion status. We used gene expression values from 26 genes (Table S2) located around and within the 8p23.1 region and we searched for any association between gene expression levels and the presence of the inversion. These analyses were carried out with the SNPassoc R package on the CEU and ASN populations, where the two 8-SNP haplotypes signature predicts the inversion status.

**Figure 3:**
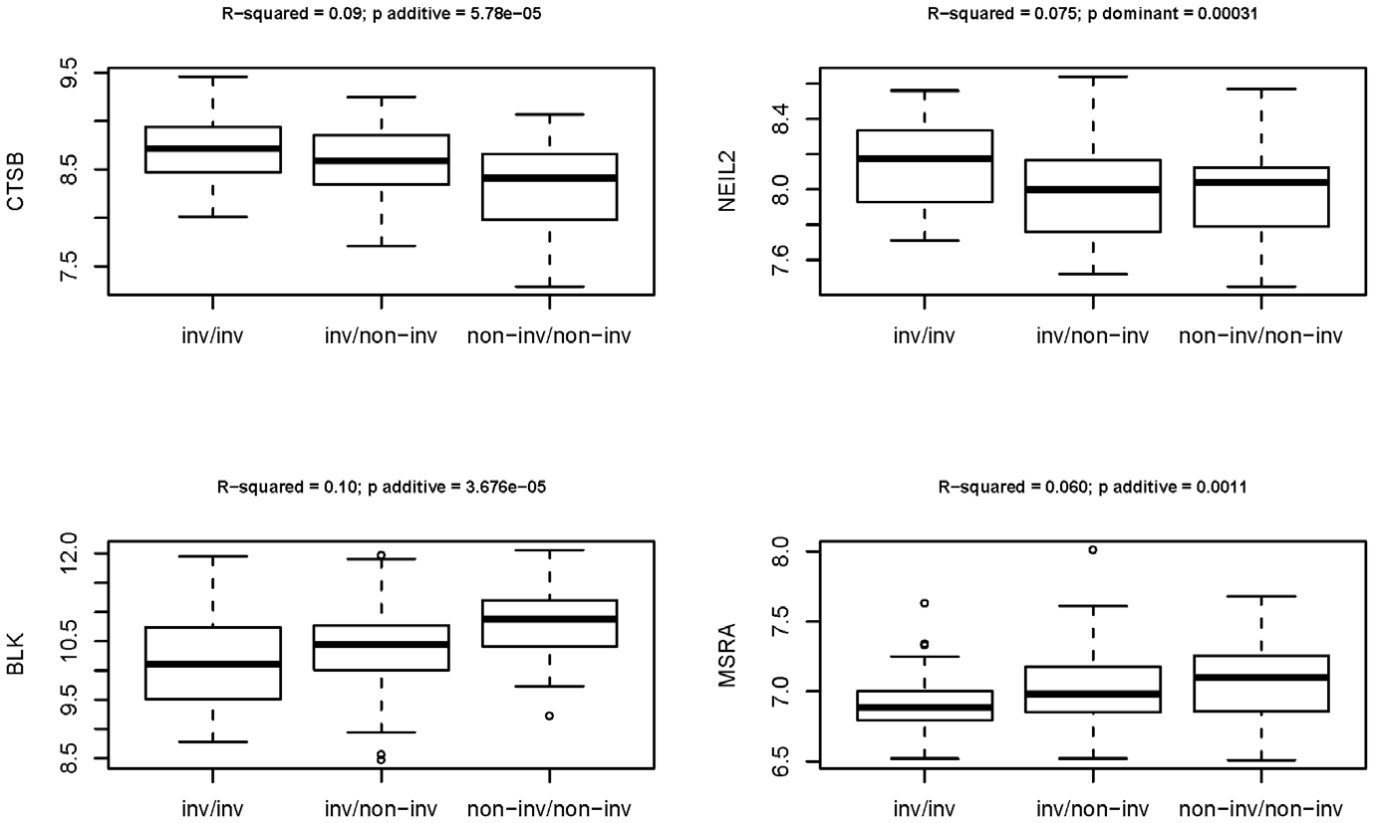
Box-plot representation for the differentially expressed CTSB, NEIL2, BLK and MSRA genes, according to the predicted inversion genotypes. The underlying distributions of expression levels for each of the three possible inversion genotypes: homozygous inverted (inv/inv), heterozygous inverted (inv/non-inv) and homozygous non-inverted (non-inv/non-inv) are shown. On the top of each panel R-squared values derived from general linear model regressions and p-values under the most significant model of inheritance are represented.

Interestingly, four genes, *NEIL2* (p = 0.0003), *MSRA* (p = 0.001), *CTSB* (p = 3.46×10^−5^) and *BLK* (p = 2.08×10^−5^) exhibited statistically significant expression level differences after Bonferroni correction for multiple testing (p<0.001) (Figure 2A). Box-plots of the different expression levels for the four genes are shown on figure 3. The model of inheritance under which the differential expression was detected was with the additive model, with the exception of *NEIL2*, which follows a dominant inheritance pattern. Under the additive model the effect of the inversion on gene expression is higher in individuals without the inversion than in the heterozygous samples, than in turn show higher levels that the homozigoulsy inverted individuals for *CTSB*, and the contrary is observed for *BLK* and *MSRA* genes. This data suggest that the inverted conformation has some effect on the expression of the genes embedded in the inverted fragment.

## Discussion

Although not as abundant as other structural variations, inversions are common genomic variants in the human genome [1], [3], [4], [31]. A recent study has described that there are at least 35 recurrent inversions that are visible by light microscopy, six of which are known to disrupt a gene [32]. An important issue emerging from these studies is the mechanism underlying such common variants. It is well known that the presence of highly identical genomic sequences (low copy repeats or SDs), arranged in opposite orientations, flanking certain regions of the genome, predispose to inversion events by non-allelic homologous recombination (NAHR). It has also been demonstrated that little requirement for long, identical homology blocks between paralogous DNA fragments is needed to produce exchanges by ectopic recombination [33]. What it is not so often taken into consideration is the presence of mitotic recombination leading to mosaicism in regions showing this type of genomic architecture. Mitotic recombination is an important mechanism under study that can complicate the interpretation of the rearrangements mediated by complex SDs, specially when the material used for study are transformed lymphoblastoid cell lines which could exhibit a high mitotic ratio. This may be the reason why several studies on the 8p23.1 region have lead to some discrepancies [4], [12]. In this regard, a recent study on mouse stem cells has shown that CNVs involving gains or losses of millions of base pairs occur frequently during mitotic cell culture divisions, supporting the idea that somatic tissues are composed of variants of the mitotic genome [34]. Similarly, a study of several tissues from different individuals has shown that somatic mosaicism is present for a subset of CNVs [35]. Finally, the analysis of concordant and discordant monozygotic twins has revealed mosaicism for CNVs, indicating somatic mosaicism for some CNV regions [36].

Our FISH results in lymphoblastoid cells prepared from Spanish control individuals as well as HapMap lymphoblastoid cell lines, points out the presence of a wide range of mosaicism regarding the 8p23.1 inversion that could have arisen during the lympholastoid cell cultures. Although previous data on this subject suggest that the effects of lymphoblastoid cells generation, as well as the effects of passage, on genotype and genetic architecture are minimal [37], it has been reported that somatic events occur during cell culture and that the mitotic events are responsible for these changes [38], [39]. It is clear that the locus analyzed here has a very complex genomic architecture due to the complex SDs it contains. Despite somatic rearrangements are a priori expected events due to the extension and high level of identity between the SDs flanking the 8p23.1 inversion polymorphism, little is known about the incidence of these events in the genome. Several studies have suggested that this phenomenon, exemplified in the case of the alpha-globins where somatic deletions encompassing these genes arise by intrachromosomal homologous exchange, is common in blood and sperm [33]. Results from Flores [40] also indicate that some cells within blood samples from normal individuals can undergo genomic rearrangements such as inversions and create genomic structural mosaicism. These findings should expand our current view about the plasticity of the genome and the homogeneity of the genetic material that we characterize in genetic studies at the SNP level.

Another aspect that arises from our study of the 8p23.1 inversion is the high frequency of the inverted allele. The possibility of these results being a misinterpretation of FISH signals due to the scarcely ∼3.5 Mb of distance between the two probes it is unlikely, since no differences regarding the levels of mosaicism were observed when the chromosomes were elongated with ethidium bromide. By means of dense SNP genotyping of the region, haplotype-based computational analyses and FISH experiments we could infer and verify the orientation status of alleles in the 8p23.1 region. Chromosomes were initially studied by FISH and surrogate markers for the inversion were identified by analyzing allelic association of 16 SNPs, which delineated two tracts of homozygosity, in a group of individuals with known status for the 8p23.1 inversion. These 16 SNP markers tag the inversion and perfectly correlate in European and Asian ancestry HapMap samples. These 16 markers were initially selected from a sample of Spanish individuals, but they were also useful to predict the inversion in individuals of CEU and ASN origin. Thus, these SNPs can be used in CEU and ASN population to tag the inversion. However, in the YRI population, which exhibits a higher SNP diversity [31], the absence of LD at these 16 surrogate markers does not allow to use them to predict the inversion in these subjects. This argues in favor of several origins for this genomic rearrangement or of an ancient origin of it. The fact that the SNP haplotype “signature” is not maintained in the YRI population is probably due to the overall higher genetic diversity carried by individuals of African descent.

We should also take into consideration that the number of SNPs found in LD with the inversion is subjected to the polymorphisms that share in common the two resources that have been used, the Illumina's HumanCNV370-Duo chip for the Spanish individuals in first instance, and subsequently the genotyping data present in the HapMap database. Thus, the 16 SNP “signature” that we have identified is based on SNPs shared by both resources, meanwhile tracts of homozygosity comprising as much as ∼100 SNPs in individuals predicted to be inverted in both populations (CEU and ASN), do overlap with the distal and proximal homozygosity tracts that we describe when we only consider the high coverage data present on HapMap phase III (data not shown). These observations do support the presence of large homozygosity tracts that are in LD with the inverted conformation of 8p23.1 region and the reliability of these 16 surrogate markers.

The analysis performed on HapMap individuals revealed that 37% of ASN population and 50% of the CEU samples were heterozygous for the inverted allele. This frequency is similar to the findings in the Spanish controls genotyped by FISH, where 50% are heterozygous for the inversion, and it is in accordance with previous published results [4], [12]. Regarding the homozygous status of the inversion, this is present in 48% of the ASN HapMap individuals, in 8% of the CEU HapMap samples and in 29% of Spanish control samples.

The fact that there is not a long stretch of SNPs along the ∼3.8 Mb region that is flanked by the SDs that involve this polymorphic inversion, and only a region of about 170 kb on both sides showing homozygosity for the SNPs, indicates that the inversion has likely been generated many generations ago, leading to many recombination events within the region. It is unknown if these stretches of homozygosity are just relics of the ancestral haplotypes in which the inversion arose or if they might have a role in facilitating its recurrence. The results presented here indicate that the recurrence of the 8p23.1 inversion is much more frequent than previously reported [9], at least in an European population, and that the current human genome reference assembly (Build 36) corresponds to the less common orientation of the 8p23.1 region, as it has been reported for several other structural variants [31].

Although inversions are generally considered as neutral variants regarding phenotype, there are several exceptions where specific genes at the breakpoints are interrupted [41]–[46]. Moreover, little is known about the effects on the regulation of the transcription of the genes close to the breakpoints where inversions occur. To investigate the possible effects on the genes of 8p23.1 region on the allelic variation of the inverted and non-inverted forms, we performed and association study of gene expression levels and the genotype of the inversion in 150 HapMap individuals. We found four genes (*NEIL2*, *MSRA*, *CTSB* and *BLK*) that show statistically significant different expression levels (p<0.0005). Two of these genes, *NEIL2* and *MSRA*, are related to repair of oxidative damage [47], [48], and *CTSB* has been suggested to play a role in Alzheimer's disease [49]. To which extent these gene expression differences can influence the function of the respective proteins and if they are directly related to the inversion of the region remains to be proved. We cannot exclude that the degree of mosaicism present in the lymphoblastoid cell lines can modulate the overall tissue-specific expression levels of these genes. In addition we still have no evidences on how this inversion accompanied by any degree of mosaicism can affect the regulation of the transcription of the genes contained in the region. *Another possibility would be that these linkage disequilibrium blocks may contain regulatory variants which are influencing the expression level of these genes*.

In summary, using dense SNP genotyping analysis of the chromosome 8p23.1 region in homozygous inverted samples previously genotyped by FISH, we have been able to describe surrogate markers that tag the 8p23.1 inversion in CEU and ASN populations. Moreover, among the 26 genes we analyzed we have observed gene expression differences in four genes depending on the alternative genomic conformation of the region. We also highlight the presence of mosaicism regarding the inversion in most of the individuals genotyped by FISH. This mosaicism will agree with the different types of discordant fosmid clones with an inverted orientation detected for this region in the analysis performed by Kidd et al. [30]. We postulate that this is a phenomenon that could also involve other regions of the genome flanked by complex or high level identity SDs and L1 transposons, as previously described [35], [50], [51]. It is currently unknown if the genomic isoforms and their variable levels of expression have consequences at the phenotypic and functional levels of the individual, deserving future investigation.

## Supporting Information

Figure S1 FISH analysis of the human chromosome 8p23.1 inversion in HapMap samples. Metaphase FISH of three HapMap individuals, NA12057 as an example of non-inverted individual; NA06993 is heterozygous for the 8p23.1 inversion and NA12815 corresponds to a homozygous inverted individual.
(1.20 MB TIF)

Figure S2 Scheme of the different sizes of 8p23.1 inversion within nine human cell lines as a result of fosmid-end cloning and sequencing. For each cell line fosmid library (ABC7 to G248) several end-sequenced fosmid clones were discordant for the mapping of the end sequences, showing an inversion with respect to the reference genome, and also showing different mapping positions. The abundance of each fosmid clone and the approximate sizes of the rearrangements are shown in Supplementary Table 1 (data extracted from Kidd et al., 2008). The filed orange-yelow boxes correspond to the segmental duplications (SD) that flank and are within the inverted polymorphic region. Nucleotide positions are in megabases (Mb).
(0.62 MB TIF)

Table S1 Sizes of large inversions detected in human cell lines in fosmid-end cloning and sequencing
(0.03 MB XLS)

Table S2 8p23.1 Gene expression levels. Genes analyzed in the association study between gene expression levels and the genotype for the 8p23.1 inversion, and their corresponding probes (http://www.sanger.ac.uk/humgen/genevar/).
(0.03 MB DOC)
