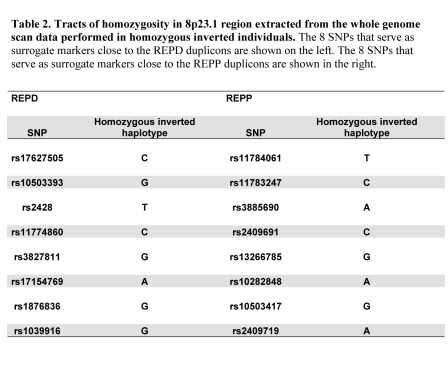# Correction: Nucleotide, Cytogenetic and Expression Impact of the Human Chromosome 8p23.1 Inversion Polymorphism

**DOI:** 10.1371/annotation/f551fde5-fde4-4485-9e9d-c94bd501a078

**Published:** 2010-06-15

**Authors:** Nina Bosch, Marta Morell, Immaculada Ponsa, Josep Maria Mercader, Lluís Armengol, Xavier Estivill

In the second column of Table 2, the top two SNPs are listed incorrectly. Please view the corrected table here: 

**Figure:**