# Supplementary figures and images for: Nucleotide, Cytogenetic and Expression Impact of the Human Chromosome 8p23.1 Inversion Polymorphism
Source: PLoS One. 2009 Dec 14;4(12):e8269. doi: 10.1371/journal.pone.0008269 (PMC2790694; doi:10.1371/journal.pone.0008269)

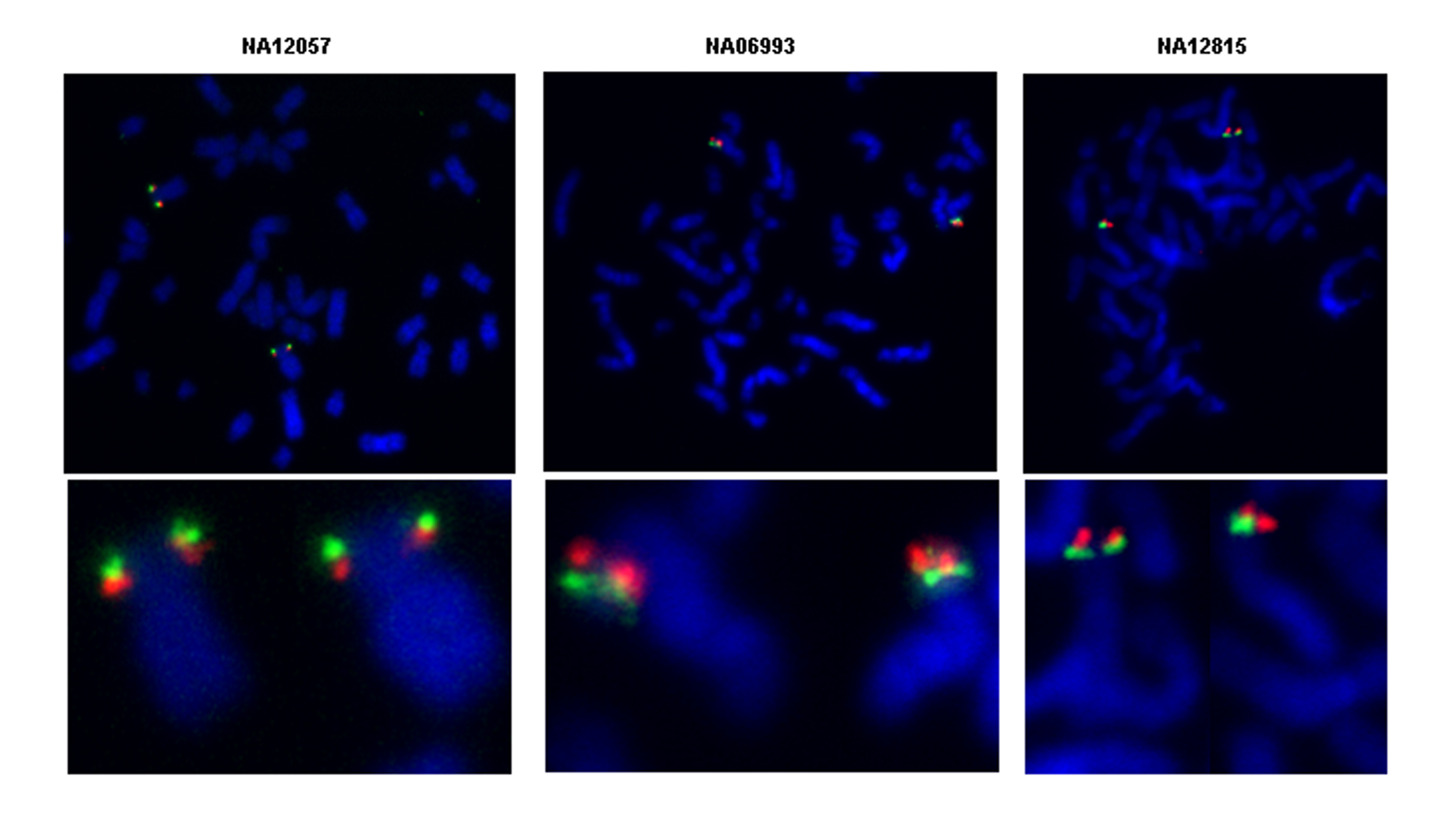

Supplement: Figure S1 — FISH analysis of the human chromosome 8p23.1 inversion in HapMap samples. Metaphase FISH of three HapMap individuals, NA12057 as an example of non-inverted individual; NA06993 is heterozygous for the 8p23.1 inversion and NA12815 corresponds to a homozygous inverted individual. (1.20 MB TIF) [file pone.0008269.s001.tif]

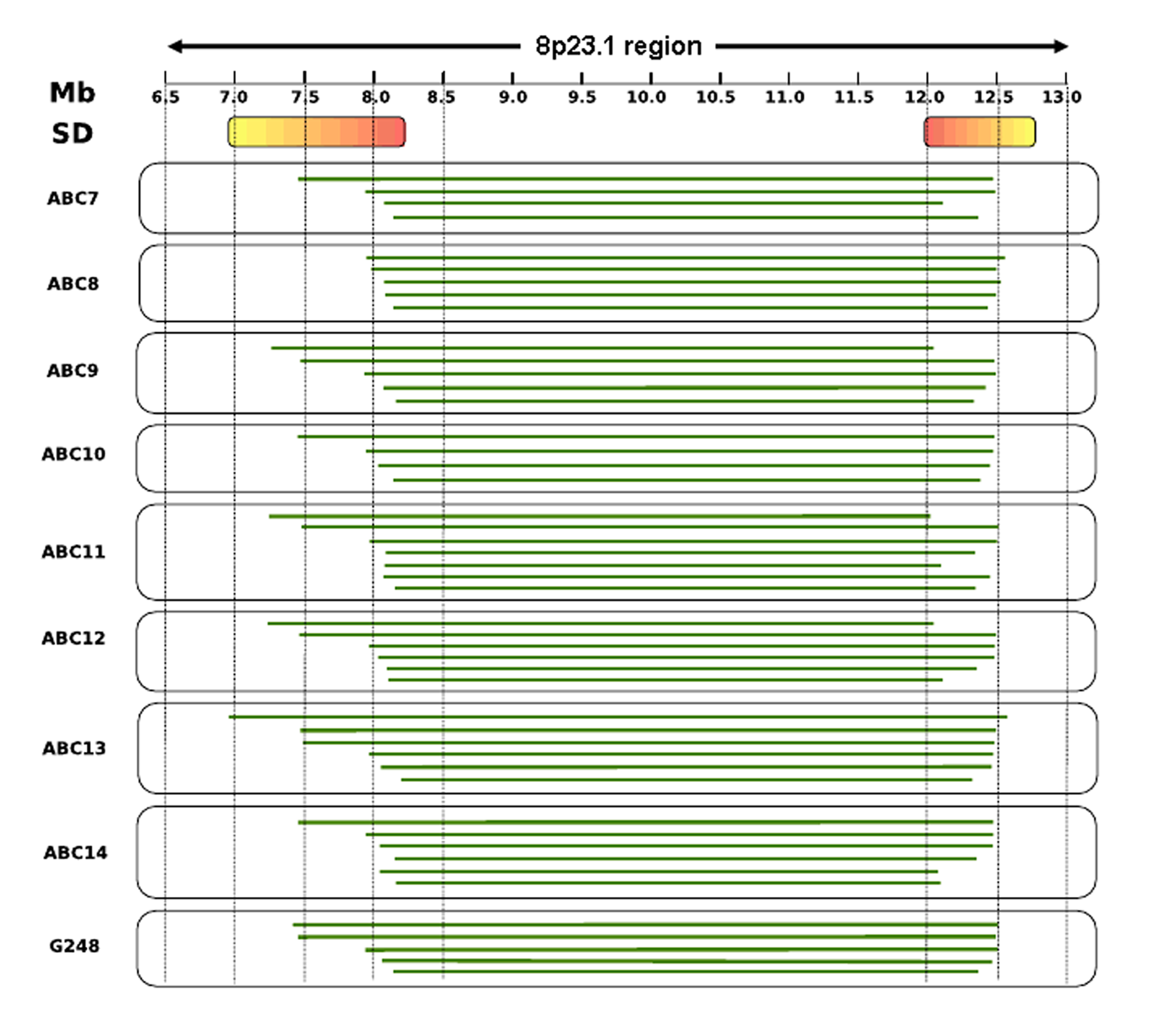

Supplement: Figure S2 — Scheme of the different sizes of 8p23.1 inversion within nine human cell lines as a result of fosmid-end cloning and sequencing. For each cell line fosmid library (ABC7 to G248) several end-sequenced fosmid clones were discordant for the mapping of the end sequences, showing an inversion with respect to the reference genome, and also showing different mapping positions. The abundance of each fosmid clone and the approximate sizes of the rearrangements are shown in Supplementary Table 1 (data extracted from Kidd et al., 2008). The filed orange-yelow boxes correspond to the segmental duplications (SD) that flank and are within the inverted polymorphic region. Nucleotide positions are in megabases (Mb). (0.62 MB TIF) [file pone.0008269.s002.tif]
