# Supplementary material for: Nucleotide, Cytogenetic and Expression Impact of the Human Chromosome 8p23.1 Inversion Polymorphism
Source: PLoS One. 2009 Dec 14;4(12):e8269. doi: 10.1371/journal.pone.0008269 (PMC2790694; doi:10.1371/journal.pone.0008269)
Supplement: Table S2 — 8p23.1 Gene expression levels. Genes analyzed in the association study between gene expression levels and the genotype for the 8p23.1 inversion, and their corresponding probes (http://www.sanger.ac.uk/humgen/genevar/). (0.03 MB DOC) [file pone.0008269.s004.doc]

**Supplemetary Table 2.** **8p23.1 Gene expression levels.** Genes analyzed in the association study between gene expression levels and the genotype for the 8p23.1 inversion, and their corresponding probes (http://www.sanger.ac.uk/humgen/genevar/)**.**
